# Maraviroc Attenuates Trauma-Hemorrhage-Induced Hepatic Injury through PPAR Gamma-Dependent Pathway in Rats

**DOI:** 10.1371/journal.pone.0078861

**Published:** 2013-10-18

**Authors:** Fu-Chao Liu, Yung-Fong Tsai, Huang-Ping Yu

**Affiliations:** 1 Department of Anesthesiology, Chang Gung Memorial Hospital, Taoyuan, Taiwan; 2 College of Medicine, Chang Gung University, Taoyuan, Taiwan; 3 Graduate Institute of Clinical Medical Sciences, Chang Gung University, Taoyuan, Taiwan; UAE University, Faculty of Medicine & Health Sciences, United Arab Emirates

## Abstract

Maraviroc is a CC-chemokine receptor 5 (CCR5) antagonist with potent antiviral and cancer preventive effects. Recent evidence suggests that the co-existence of CCR5 in various cell types is involved in inflammation. However, the effects that CCR5 antagonists produce in trauma-hemorrhage remain unknown. The peroxisome proliferator-activated receptor gamma (PPAR_γ_) pathway exerts anti-inflammatory effects in injury. In this study, we hypothesized that maraviroc administration in male rats, after trauma-hemorrhage, decreases cytokine production and protects against hepatic injury through a PPAR_γ_-dependent pathway. Male Sprague-Dawley rats underwent trauma-hemorrhage (mean blood pressure maintained at approximately 35-40 mmHg for 90 minutes), followed by fluid resuscitation. During resuscitation, a single dose of maraviroc (3 mg/kg, intravenously) with and without a PPAR_γ_ antagonist GW9662 (1 mg/kg, intravenously), GW9662 or vehicle was administered. Plasma alanine aminotransferase (ALT) with aspartate aminotransferase (AST) concentrations and various hepatic parameters were measured (n=8 rats/group) at 24 hours after resuscitation. The results showed that trauma-hemorrhage increased hepatic myeloperoxidase activity, intercellular adhesion molecule-1 and interleukin-6 levels, and plasma ALT and AST concentrations. These parameters were significantly improved in the maraviroc-treated rats subjected to trauma-hemorrhage. Maraviroc treatment also increased hepatic PPAR_γ_ expression compared with vehicle-treated trauma-hemorrhaged rats. Co-administration of GW9662 with maraviroc abolished the maraviroc-induced beneficial effects on the above parameters and hepatic injury. These results suggest that the protective effect of maraviroc administration on alleviation of hepatic injury after trauma-hemorrhage, which is, at least in part, through PPAR_γ_-dependent pathway.

## Introduction

Trauma-hemorrhage can induce massive pro-inflammatory mediators production, such as chemokines and cytokines [1,2]. Despite fluid resuscitation, trauma-hemorrhage induces tissue and organ damage, including the liver. Hepatic dysfunction reflects the severity of tissue injury and is associated with poor outcome following trauma-hemorrhage [3].

The peroxisome proliferator-activated receptor gamma (PPAR_γ_) is expressed in various cells including endothelial cells, smooth muscle cells, macrophages, monocytes, and kupffer cells and involves in the regulation of inflammatory responses [4,5]. Previous studies have shown that PPAR_γ_ signalling pathways play important roles in animal models of ischemia/reperfusion and inflammation [6-8]. PPAR_γ_ also plays a key role in shock-induced myocardial, lung and hepatic injuries [9,10]. The PPAR_γ_ affects pro-inflammatory cytokines production and chemotactic events in response to injury [8,9,12]. In addition, the PPAR_γ_ has a pivotal role in neutrophils migration to undergo chemotaxis [13,14]. Previous studies have also shown that activation of the PPAR_γ_ attenuates the overproduction of cytokines, adhesion molecules, and neutrophil accumulation after trauma-hemorrhage [9,10,15].

Maraviroc, an antagonist of CC-chemokine receptor 5 (CCR5), is a potent antiretroviral drug used to treat human immunodeficiency virus (HIV) infection [16,17] and prevents development of cancer cells in animal studies [18]. Previous evidence suggests the presence of CCR5 in various cell types involved in inflammation [19]. CCR5 deficiency mice have lower inflammatory pain under chemical or inflammatory stimuli [20]. Recent studies have shown that maraviroc can protect against organ injury following allograft [21]. However, maraviroc may exert anti-inflammatory effects, though its effects in trauma-hemorrhage remain unknown. Furthermore, previous studies have shown that an increase in PPAR_γ_ activity improves liver function following trauma-hemorrhage or ischemia injury [6,9,15]. It is implied that PPAR_γ_ may play a role in maraviroc-mediated hepatoprotection following trauma-hemorrhage. We hypothesized that the beneficial effects of maraviroc following trauma-hemorrhage are mediated via a PPAR_γ_-related pathway. To test this hypothesis, animals were treated with maraviroc alone and in combination with the PPAR_γ_ antagonist GW9662 after trauma-hemorrhage. The effects of these treatments were then examined with respect to hepatic injury as well as hepatic myeloperoxidase (MPO) activity, intercellular adhesion molecule-1 (ICAM-1), interleukin-6 (IL-6), and PPAR_γ_ levels following trauma-hemorrhage.

## Materials and Methods

### Animals

Adult male Sprague-Dawley strain rats were used in this study. The rats were obtained from the National Science Council Experimental Animal Center. All animal experiments were performed according to the guidelines of the *Animal Welfare Act* and *The Guide for Care and Use of Laboratory Animals* from the National Institutes of Health. All procedures and protocols were approved by the Institutional Animal Care and Use Committee of Chang Gung Memorial Hospital.

### Rat Trauma-Hemorrhage Model

A non-heparinized rat model of trauma-hemorrhage was used in this study [22]. Thirty-six male Sprague-Dawley rats (275–325 g) were randomly assigned to 6 groups (n=6/group). Initial studies examined trauma-hemorrhage, with the groups receiving maraviroc (0, 0.3, 1, 3, or 5 mg/kg); sham groups were also included. In addition, forty-eight male Sprague-Dawley rats were randomly divided into 6 separate groups (n=8/group). All animals were placed in the animal house individually in cages with air-conditioned (humidity 70–75%), controlled temperature (24–25°C) and lighting (light- dark cycle every 12 hours: lights on 06:00 to 18:00). Basal diet and water was provided and allowed at least 1 week to adapt to the environment. Before initiation of the experiment, male Sprague- Dawley rats were fasted overnight but allowed free water access. Trauma-hemorrhage and resuscitation was then performed as described previously [22]. In brief, rats were anesthetized by isoflurane inhalation, and a 5-cm midline laparotomy was performed to induce soft tissue trauma. The abdominal wound was then closed in layers. Polyethylene catheters (PE-50; Becton Dickinson & Co., Sparks, MD) were placed in both femoral arteries and the right femoral vein from bilateral inguinal incision wounds (about 0.5 cm in length), and the bilateral inguinal incision sites were then closed. The wounds were bathed with 1% lidocaine (Elkins-Sinn Inc., Cherry Hill, NJ) throughout the operative procedure to reduce postperative pain. The rats were allowed to awaken, after which they were bled rapidly within 10 minutes to a mean arterial pressure of 35 to 40 mmHg. This level of hypotension was maintained until the animals could no longer maintain a mean arterial pressure of 40 mmHg unless some fluid in the form of Ringer’s lactate was administered. This time was defined as maximum bleed-out. After the maximal bleed-out, mean arterial pressure was maintained between 35 to 40 mmHg until 40% of the maximal bleed-out volume was returned in the form of Ringer’s lactate solution (about 90 minutes from the onset of bleeding). The rats were then resuscitated with four times the volume of the shed blood with Ringer’s lactate for 60 minutes. Thirty minutes before the end of the resuscitation period, the rats received maraviroc (3 mg/kg, intravenously), maraviroc plus the PPAR_γ_ antagonist GW9662 (1 mg/kg, intravenously at the beginning of resuscitation), GW9662, or an equal volume of the vehicle (about 0.2 mL, DMSO). After resuscitation, the catheters were removed, the vessels ligated, and the skin incisions closed with sutures. Sham-operated animals underwent all operative procedures, but neither hemorrhage nor resuscitation was performed. Vehicle or maraviroc was also administered in sham-operated rats after catheters were placed. The animals were humanely killed at 24 hours after the end of resuscitation or sham operation. In the experiment under review, there were 8 rats in each group.

### Measurement of Hepatic Injury

At 24 hours after trauma-hemorrhage or sham operation, blood samples with heparin were obtained and plasma was separated by centrifugation. Hepatic injury was determined by measuring plasma levels of AST and ALT using a colorimetric analyzer (Dri-Chem 3000; Fuji Photo Film Co., Tokyo, Japan).

### Measurement of MPO Activity

MPO activity in homogenates of liver tissues was determined as described previously [22]. Frozen tissue samples were thawed and suspended in phosphate buffer (pH 6.0) containing 0.5% hexadecyltrimethylammonium bromide (Sigma, St. Louis, MO). The samples were sonicated on ice, centrifuged at 12,000 g for 15 minutes at 4° C, and an aliquot was transferred into phosphate buffer (pH 6.0) containing 0.167 mg/mL o-dianisidine hydrochloride and 0.0005% hydrogen peroxide (Sigma, St. Louis, MO). The change in absorbance at 460 nm was measured spectrophotometrically for 5 minutes. MPO activity was calculated using a standard curve that was generated using human MPO (Sigma, St. Louis, MO), and values were normalized to protein concentration.

### Measurement of ICAM-1 and IL-6 Levels

The liver tissues were homogenized in PBS (1:10 weight:volume; pH 7.4) containing protease inhibitors (Complete Protease Inhibitor Cocktail; Boehringer, Mannheim, Germany). The homogenates were centrifuged at 2,000 g for 20 minutes at 4°C and the supernatant was analyzed for the presence of ICAM-1 and IL-6 using ELISA kits (R&D, Minneapolis, MN) according to the manufacturer’s instructions and as described previously [22]. An aliquot of the supernatant was used to determine protein concentration by the Bio-Rad DC Protein Assay (Bio-Rad, Hercules, CA).

### Western Blot Assay

Rat liver tissues were homogenized in a buffer as described previously [22]. The homogenates were centrifuged at 12,000 g for 15 minutes at 4°C, analyzed using SDS-PAGE, and the proteins were then transferred to nitrocellulose membranes. The membranes were incubated with antibodies for PPAR_γ_ protein (1:1000 dilution; Cell Signaling Technology, Beverly, MA) or GAPDH (1:5000 dilution; Abcam, Cambridge, MA) overnight at 4°C. The membranes were incubated with horseradish peroxidase-conjugated goat anti-rabbit antibody or goat anti-mouse antibody for 1.5 hours at room temperature. After the final washing, blots were probed using enhanced chemiluminescence (Amersham, Piscataway, NJ) and autoradiographed.

### Statistical Analysis

For statistical analysis we used the InStat 3.0 biostatistics program (Graph Pad Software Inc., San Diego, CA). Results are presented as mean ± standard error of the mean (SEM). The data were analyzed using one-way analysis of variance (ANOVA) and the Tukey test, and differences were considered significant at *p*≤0.05.

## Results

### Dose-Response Effects of Maraviroc on Plasma AST and ALT Levels

**Figure 1:**
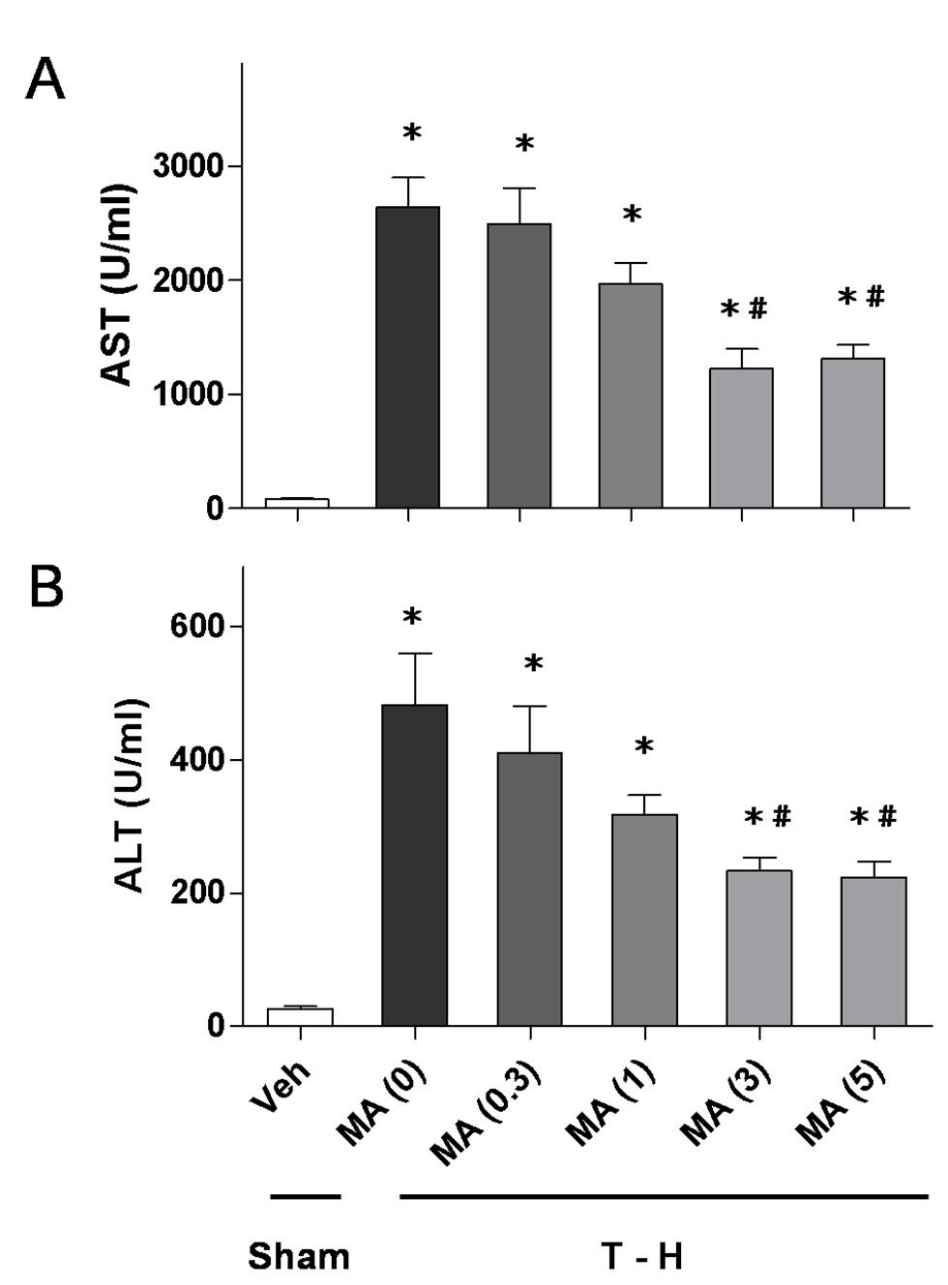
Dose-dependent responses to maraviroc treatment of plasma AST (A) and ALT (B) in rats at 24 hours after sham operation (sham) or trauma-hemorrhage and resuscitation (T-H). Animals were treated with maraviroc (MA) at doses of 0, 0.3, 1, 3, or 5 mg/kg. Data are shown as the mean ± SEM. n=6 rats in each group. ^*^ *p*<0.05 compared with sham; ^#^ *p*<0.05 compared with T-H + MA (0 mg/kg).

As shown in Figures 1A and 1B, trauma-hemorrhage was related to a significant increase in plasma AST and ALT levels at 24 h after resuscitation. Administration of maraviroc at a dose of 0.3, 1, 3, or 5 mg/kg was used to evaluate the effects of maraviroc on the attenuation of hepatic injury after trauma-hemorrhage. As shown in Figure 1, there was a diminished benefit when maraviroc was administered at the dose of 0.3 or 1 mg/kg. The effects of maraviroc were equivalent when administered at a dose of 3 or 5 mg/kg.

### Alteration in Plasma AST and ALT Levels

**Figure 2:**
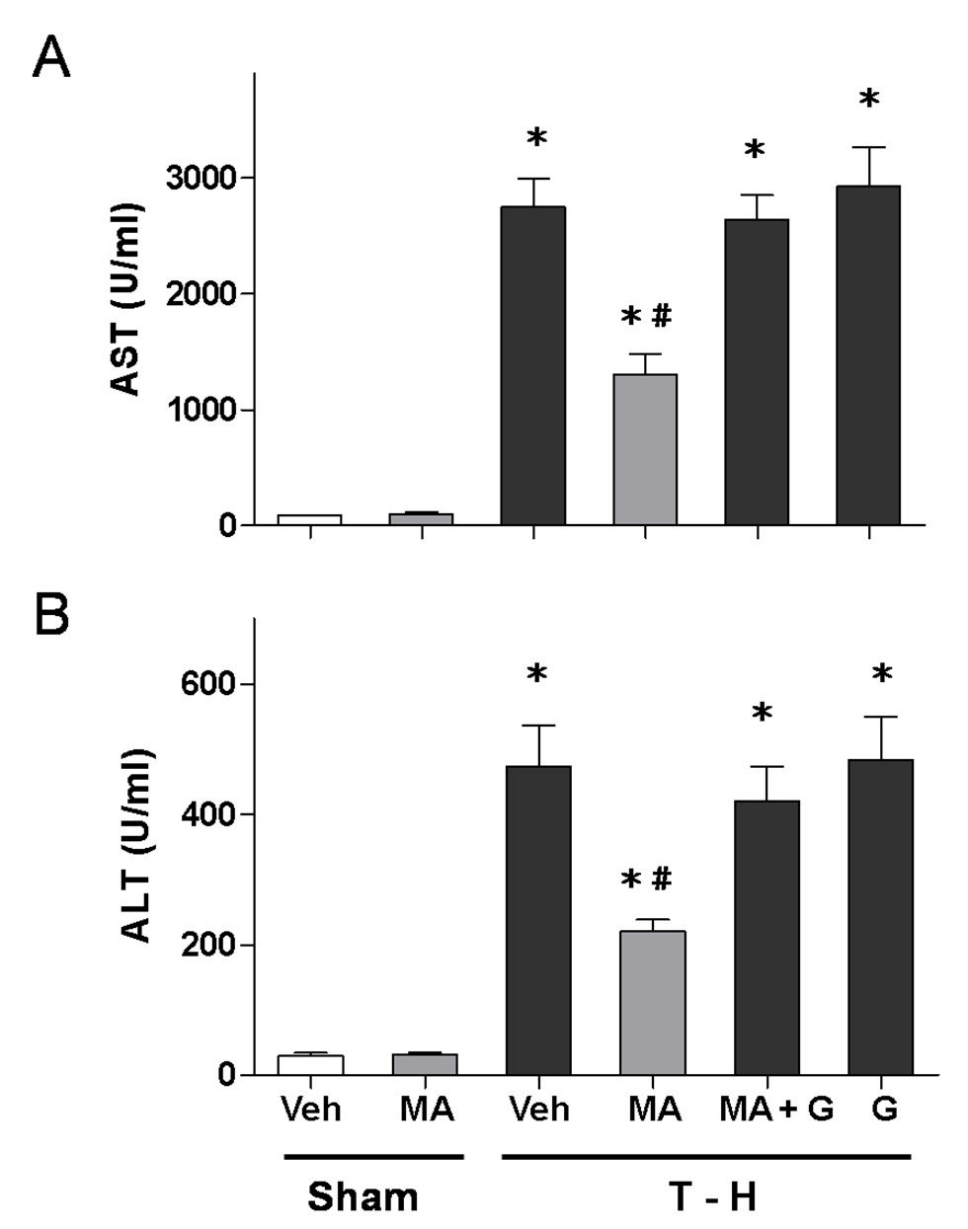
Effect of maraviroc treatment on plasma AST (A) and ALT (B) in rats at 24 hours after sham operation (Sham) or trauma-hemorrhage and resuscitation (T-H). Animals were treated with either vehicle (Veh), maraviroc (MA), maraviroc in combination with GW9662 (MA+G) or GW9662 (G). Data are shown as mean ± SEM of 8 rats in each group. ^*^ *p*<0.05 compared to Sham; ^#^ *p*<0.05 compared to T-H+Veh, T-H+MA+G and T-H+G.

As shown in Figures 2A and 2B, no significant difference in plasma AST and ALT levels was observed between vehicle- and maraviroc-treated sham groups. At 24 hours after trauma-hemorrhage, there were significant increases in plasma AST and ALT levels. Maraviroc (3 mg/kg) treatment attenuated the trauma-hemorrhage-induced increase in plasma AST and ALT levels. To determine whether the salutary effects of maraviroc in attenuating hepatic injury after trauma-hemorrhage were mediated via a PPAR_γ_-mediated activity, a group of maraviroc-treated trauma-hemorrhage rats were administrated with the PPAR_γ_ antagonist GW9662. The results indicated that administration of the PPAR_γ_ antagonist GW9662 prevented the maraviroc-induced decrease in plasma AST and ALT levels.

### Alteration in Hepatic MPO Activity

**Figure 3:**
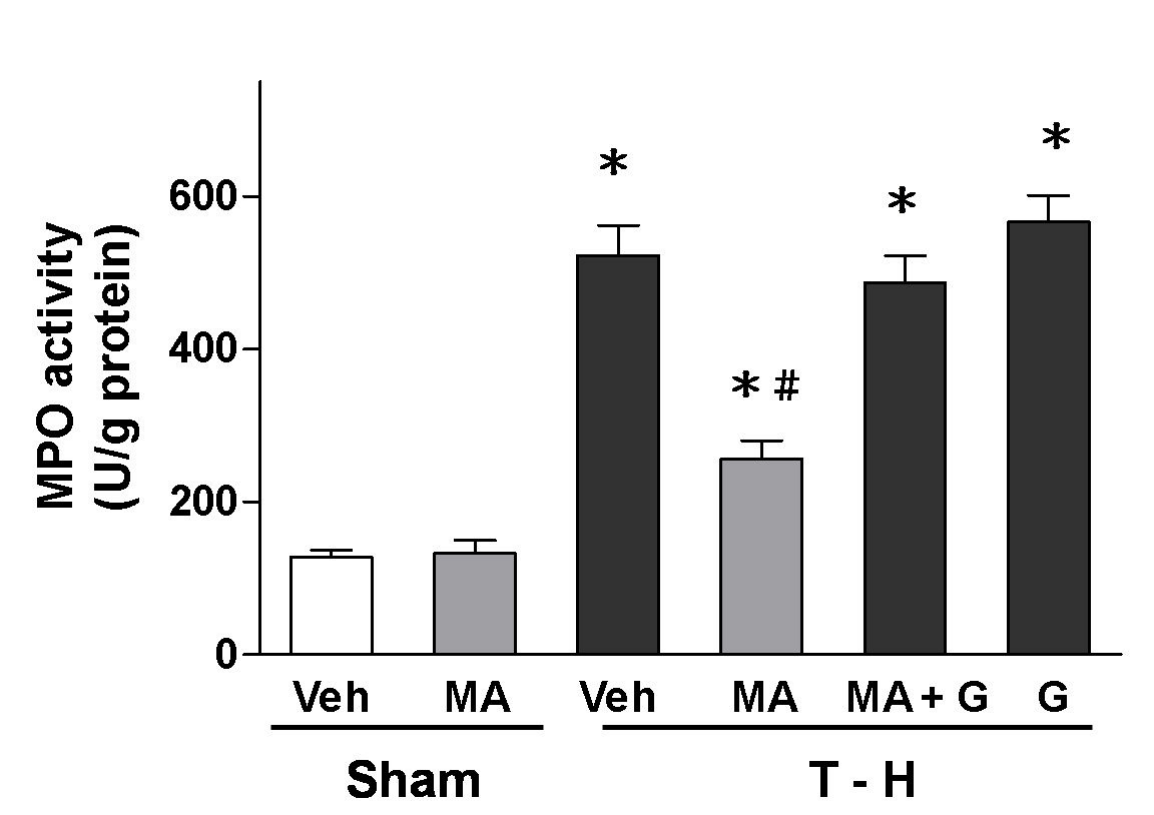
Effect of maraviroc treatment on hepatic MPO activity in rats at 24 hours after sham operation (Sham) or trauma-hemorrhage and resuscitation (T-H). Animals were treated with either vehicle (Veh), maraviroc (MA), maraviroc in combination with GW9662 (MA+G) or GW9662 (G). Data are shown as mean ± SEM of 8 rats in each group. ^*^ *p*<0.05 compared to Sham; ^#^ *p*<0.05 compared to T-H+Veh, T-H+MA+G, and T-H+G.

Hepatic MPO activity in sham or trauma-hemorrhaged animals, with and without maraviroc treatment, was shown in Figure 3. In sham-operated rats, maraviroc did not alter hepatic MPO activity. Trauma-hemorrhage resulted in a significant increase in hepatic MPO activity in vehicle-treated animals. Maraviroc treatment attenuated the increase in hepatic MPO activity. Furthermore, administration of the PPAR_γ_ antagonist GW9662 prevented the maraviroc-mediated attenuation of hepatic MPO activity after trauma-hemorrhage.

### Alteration in Hepatic ICAM-1 Concentrations

**Figure 4:**
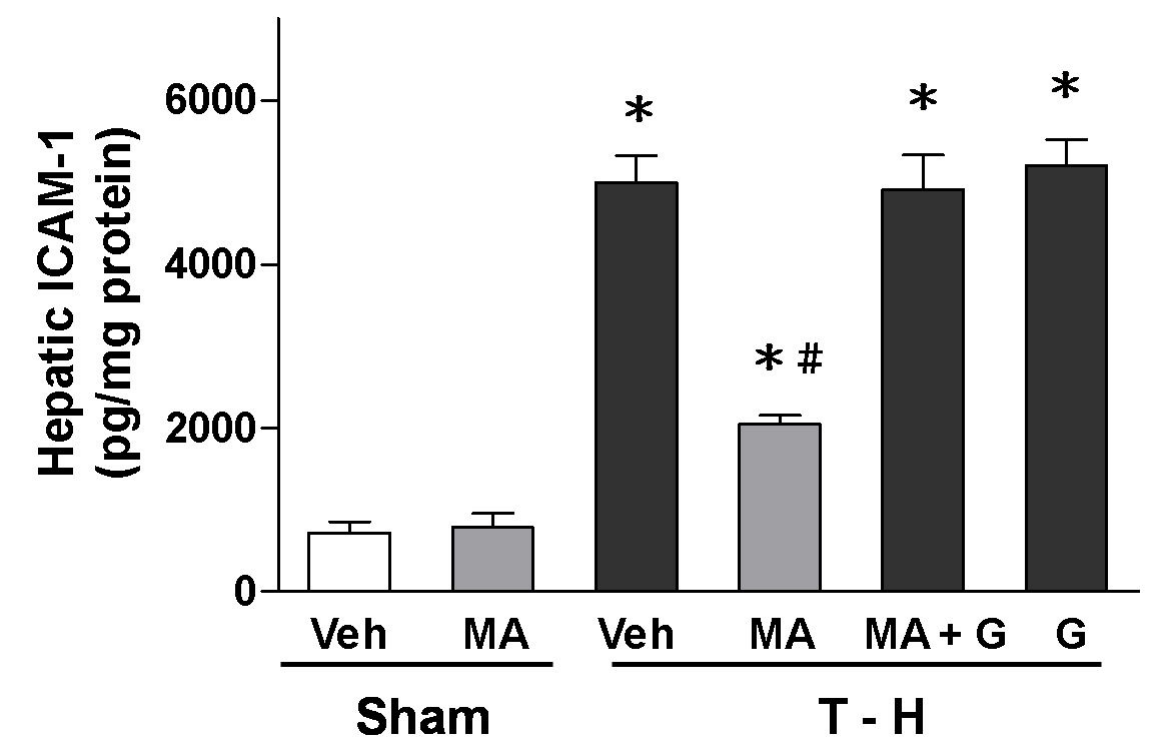
ICAM-1 levels in the liver in rats after sham operation (Sham) or trauma-hemorrhage and resuscitation (T-H). Animals were treated with vehicle (Veh), maraviroc (MA), maraviroc in combination with GW9662 (MA+G) or GW9662 (G). Data are shown as mean ± SEM of 8 rats in each group. ^*^ *p*<0.05 compared to Sham; ^#^ *p*<0.05 compared to T-H+Veh, T-H+MA+G, and T-H+G.

Trauma-hemorrhage significantly increased ICAM-1 concentrations in the liver (Figure 4). Treatment with maraviroc attenuated the trauma-hemorrhage-induced increase in ICAM-1 concentrations. Co-administration of the PPAR_γ_ antagonist GW9662 with maraviroc prevented the maraviroc-induced reduction in ICAM-1 concentrations.

### Alteration in Hepatic IL-6 Levels

**Figure 5:**
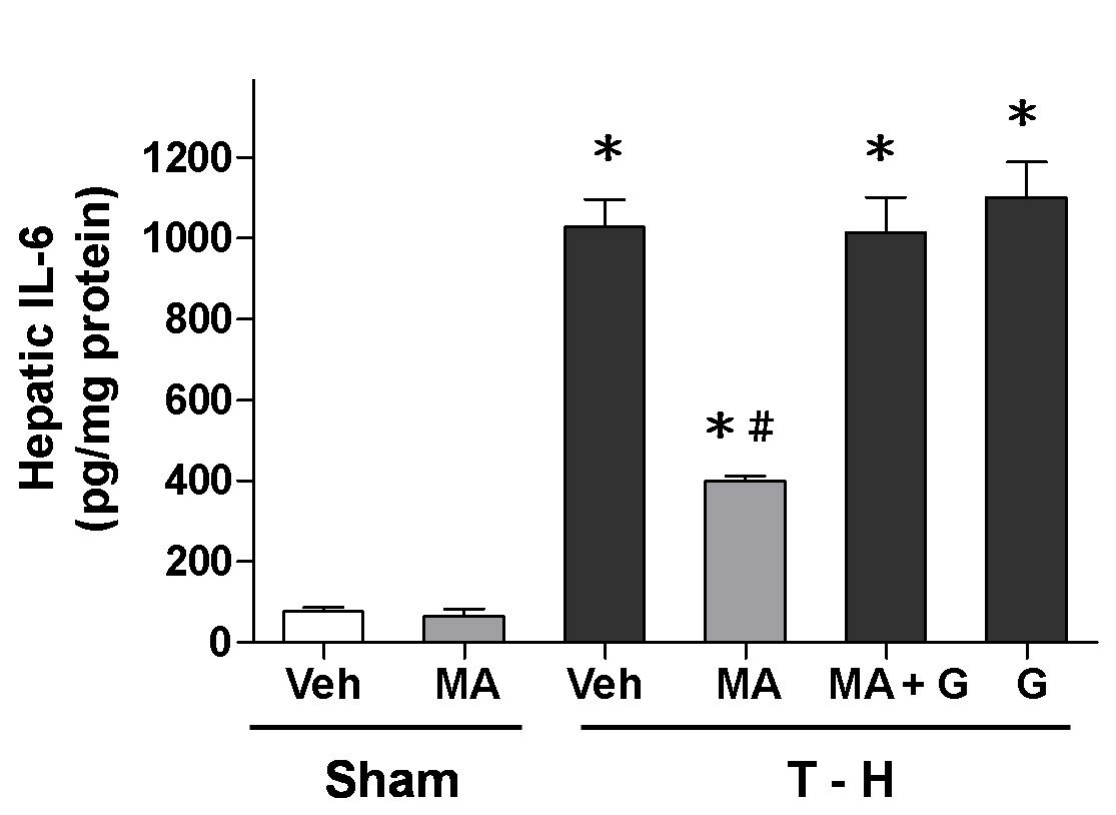
Effect of maraviroc treatment on hepatic IL-6 levels in rats at 24 hours after sham operation (Sham) or trauma-hemorrhage and resuscitation (T-H). Animals were treated with either vehicle (Veh), maraviroc (MA), maraviroc in combination with GW9662 (MA+G) or GW9662 (G). Data are shown as mean ± SEM of 8 rats in each group. ^*^ *p*<0.05 compared to Sham; ^#^ *p*<0.05 compared to T-H+Veh, T-H+MA+G, and T-H+G.

There was no significant difference in hepatic IL-6 levels between the vehicle- and maraviroc-treated sham groups (Figure 5). Trauma-hemorrhage significantly increased hepatic IL-6 levels in vehicle-treated rats compared with sham animals. The increase in hepatic IL-6 levels was reduced by maraviroc treatment, and the maraviroc-mediated reduction in IL-6 levels was abolished by PPAR_γ_ antagonist GW9662 co-administration.

### Hepatic PPAR_γ_ Protein Expression

**Figure 6:**
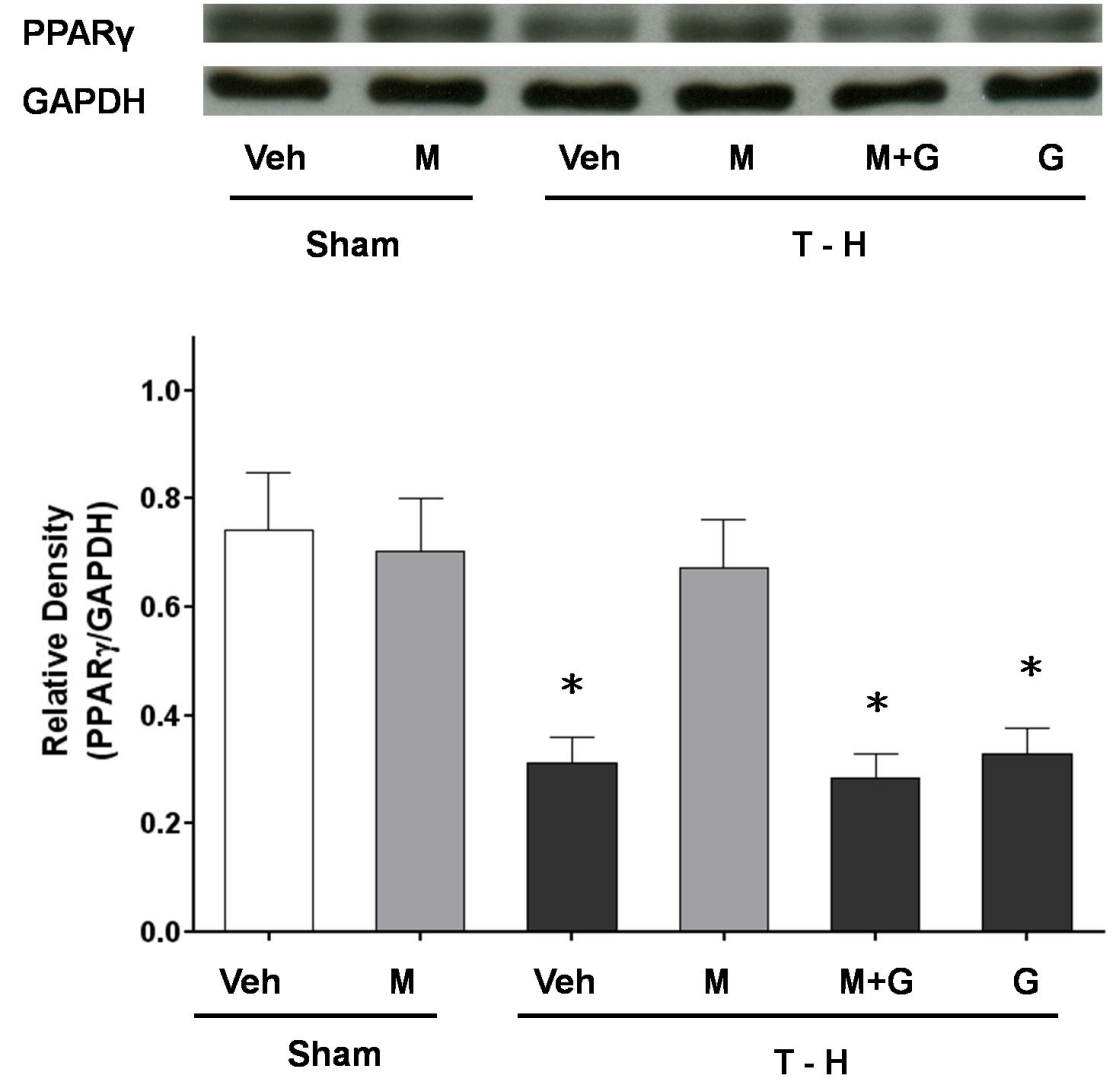
Hepatic PPAR_γ_ protein expressions from sham-operated animals receiving vehicle (Sham+Veh; lane 1) or maraviroc (Sham+MA; lane 2), trauma-hemorrhage animals receiving vehicle (T-H+Veh; lane 3), maraviroc (T-H+MA; lane 4), maraviroc and GW9662 (T-H+MA+G; lane 5) or GW9662 (T-H+G; lane 6). Blots were reprobed for GAPDH as a control for equal protein loading in all lanes. The bands were analyzed using densitometry, and the values are presented as mean ± SEM for 8 rats in each group. ^*^ *p*<0.05 versus all other groups.

Hepatic PPAR_γ_ expression in sham or trauma-hemorrhaged animals, with and without maraviroc treatment, was shown in Figure 6. In sham-operated rats, maraviroc did not alter hepatic PPAR_γ_ protein expression. Trauma-hemorrhage resulted in a significant decrease in hepatic PPAR_γ_ protein expression in vehicle-treated animals. Maraviroc treatment attenuated the decrease in hepatic PPAR_γ_ protein expression. Furthermore, administration of the PPAR_γ_ antagonist GW9662 prevented the maraviroc-mediated attenuation of hepatic PPAR_γ_ protein expression after trauma-hemorrhage.

## Discussion

In present study, we sought to determine whether PPAR_γ_-dependent pathways play an important role in maraviroc-mediated hepatoprotection following trauma-hemorrhage. The salutary effects of maraviroc at doses of 3 mg/kg have been evaluated in hepatic injury after trauma-hemorrhage. Our results indicated that administration of maraviroc (3 mg/kg) attenuated trauma-hemorrhage-induced hepatic injury. Twenty-four hours after trauma-hemorrhage, hepatic MPO activity, ICAM-1 and IL-6 levels were markedly increased in male rats. Administration of maraviroc (3 mg/kg) during resuscitation attenuated the increases in those parameters. Administration of maraviroc also prevented the trauma-hemorrhage-induced decrease in PPAR_γ_ expression. Furthermore, our findings indicated that administration of the PPAR_γ_ antagonist GW9662 along with maraviroc abolished the maraviroc-induced hepatoprotection in rats subjected to trauma-hemorrhage. These studies collectively suggest that the salutary effects of maraviroc seem to be mediated via a PPAR_γ_-dependent pathway.

The liver is an important organ that plays critical roles in the body. Liver injury following trauma-hemorrhage can lead to serious life threatening conditions. Previous studies have shown that hepatic injury is associated with increased neutrophil accumulation [22,23]. The infiltration of neutrophils is accompanied by increased expression of adhesion molecules and cytokines [22,23]. MPO activity is an indicator of neutrophil activation, and it has been correlated with tissue ICAM-1 expression after trauma-hemorrhage [2,22,24]. Our results showed that trauma-hemorrhage resulted in a significant increase in hepatic ICAM-1 levels, which was accompanied by elevated hepatic MPO activity. However, MPO activity and ICAM-1 levels were attenuated in maraviroc-treated trauma-hemorrhaged rats. Maraviroc is an antiretroviral agent and often used in the treatment of HIV infection [17,25]. CCR5 receptor antagonists, particularly maraviroc, possess anti-inflammatory properties [26,27]. Previous studies have shown that maraviroc can inhibit fMLP-induced chemotactic activity of monocytes, macrophages and dendritic cells [19] in vitro and against organ injury following allograft [21]. Recent studies have shown that CCR5-deficient mice attenuates chemical or inflammatory stimuli [20]. However, little is known about the role of maraviroc in trauma-hemorrhage.

IL-6 plays a important role in neutrophil infiltration following tissue hypoxia or organ injury [24,28]. In addition, IL-6 is required for the expression of adhesion molecules and chemokines following trauma-hemorrhage [22]. In this study, hepatic IL-6 levels were attenuated in the rats treated with maraviroc after trauma-hemorrhage. The ability of maraviroc to modulate expression of inflammatory cytokine (IL-6) and adhesion molecule (ICAM-1) suggests a role for maraviroc in the regulation of hepatic injury following trauma-hemorrhage.

PPAR_γ_, a member of the nuclear hormone receptor superfamily, was known originally to play a key role in adipocyte differentiation and glucose homeostasis [5]. The PPAR_γ_ is now shown to play a pivotal role in the cell survival and organ protection [8,15]. Previous studies have shown that an endogenous PPAR-gamma agonist, 15-deoxy-Δ^12,14^-prostaglandin J_2_ (15d-PGJ_2_), can reduce inflammation-induced neutrophil migration in mesenteric tissues [13] and attenuate liver injury after hemorrhagic shock in rat model [11,29]. A growing body of evidences indicate that PPAR-gamma agonists (Pioglitazone, Rosiglitazone) attenuate myocardial [30], hepatic [6] and renal [31] ischemia-reperfusion injury and protect against traumatic brain injury and spinal cord injury in rodent models [32]. The results suggest that administration of PPAR-gamma agonists may increase PPAR-gamma activity and create protective effect. Recent studies suggest that PPAR_γ_ activation can decrease vascular inflammation through inhibition of ICAM-1 expression [33]. 

GW9662 (2-chloro-5-nitrobenzanilide) is a potent PPAR_γ_ antagonist [34]. Several studies have reported that inhibition of the PPAR_γ_ pathway with the GW9662 decreases the survival of cells and increases the degree of organs injury following trauma-hemorrhage [11,29]. Our finding showed that trauma-hemorrhage was accompanied by a decrease in hepatic PPAR_γ_ activation. The depressed PPAR_γ_ following trauma-hemorrhage was restored by administration of maraviroc after trauma-hemorrhage. However, the increase in PPAR_γ_ by maraviroc after trauma-hemorrhage was abolished by co-administration of GW9662. These results thus indicate that the salutary effects of maraviroc on hepatic function after trauma-hemorrhage are in part mediated by a PPAR_γ_-dependent pathway.

From this study, maraviroc activated hepatic PPAR_γ_ and decreased hepatic ICAM-1 and IL-6 levels and neutrophil activity following trauma-hemorrhage. The finding that maraviroc can attenuate hepatic dysfunction and inflammatory responses when administered therapeutically, i.e. during the resuscitation phase, may be important.  These data may have translational significance and clinical relevance. Our findings have also provided insights into the mechanism by which maraviroc therapeutically attenuates hepatic dysfunction and inflammatory responses following trauma-hemorrhage.

The trauma-hemorrhagic accident is usually sudden and unexpected. Earlier treatment may produce better therapeutic effect. In clinical condition, the trauma-hemorrhagic patients usually receive the medical treatment during the resuscitation period. In our trauma-hemorrhage and resuscitation model, the rats were resuscitated for 60 minutes following trauma-hemorrhage and maraviroc was given 30 minutes before the end of resuscitation. The timing of maraviroc administration is according to our previous studies and might be reasonable for clinical condition [2,22,24]. However, it remains unknown if maraviroc is administered at the onset of resuscitation or before resuscitation. The clinical implication will be largely limited to prophylactic use.

In conclusion, our study indicates that maraviroc administration ameliorates hepatic injury and production of pro-inflammatory mediators after trauma-hemorrhage. Blockade of PPAR_γ_ activation abolishes the salutary effects of maraviroc in the liver following trauma-hemorrhage. Our findings provide evidence that maraviroc-mediated hepatoprotection is, in part, mediated via a PPAR_γ_-dependent pathway after trauma hemorrhage. Maraviroc may be a novel adjunct for improving depressed hepatic function under adverse circulatory conditions.
